# TTK Protein Kinase promotes temozolomide resistance through inducing autophagy in glioblastoma

**DOI:** 10.1186/s12885-022-09899-1

**Published:** 2022-07-18

**Authors:** Jian Yu, Ge Gao, Xiangpin Wei, Yang Wang

**Affiliations:** Department of Neurosurgery, The First Affiliated Hospital of USTC, Division of Life Sciences and Medicine, University of Science and Technology of China, 17 Lujiang Road, Hefei, 230001 Anhui China

**Keywords:** TTK, Temozolomide, Glioblastoma, Autophagy, Resistance

## Abstract

**Background:**

Temozolomide (TMZ) resistance remains the main therapy challenge in patients with glioblastoma multiforme (GBM). TTK Protein Kinase (TTK) contributes to the radioresistance and chemoresistance in many malignancies. However, the role of TTK in the TMZ resistance of GBM cells remains unknown.

**Methods:**

The expression of TTK was measured by western blot. The proliferation of GBM cells was assessed through MTT assay and clonogenic assay. Cell apoptosis was evaluated using western blot. LC3B puncta were detected using immunohistochemistry staining. The mouse xenograft model was used to investigate the role of TTK in vivo.

**Results:**

Knockdown of TTK increased the sensitivity of GBM cells to TMZ treatment, while overexpression of TTK induced TMZ resistance. Two specific TTK inhibitors, BAY-1217389 and CFI-402257, significantly inhibited GBM cell proliferation and improved the growth-suppressive effect of TMZ. In addition, the knockdown of TTK decreased the autophagy levels of GBM cells. Inhibition of TTK using specific inhibitors could also suppress the autophagy process. Blocking autophagy using chloroquine (CQ) abolished the TMZ resistance function of TTK in GBM cells and in the mouse model.

**Conclusions:**

We demonstrated that TTK promotes the TMZ resistance of GBM cells by inducing autophagy in vitro and in vivo. The use of a TTK inhibitor in combination with TMZ might help to overcome TMZ resistance and improve therapy efficiency in GBM.

**Supplementary Information:**

The online version contains supplementary material available at 10.1186/s12885-022-09899-1.

## Background

Glioblastoma multiforme (GBM) is one of the most common and malignant tumors in the brain worldwide, accounting for 48.3% of primary malignant brain tumors [1, 2]. Although extensive efforts have been done to improve the therapeutic effectiveness of GBM, patients bearing GBM have a median overall survival (OS) of 14–17 months, with only 43% of them surviving for 2 years [3]. Except for maximal surgical resection and radiotherapy, alkylating drug temozolomide (TMZ) based chemotherapy also serves as a standard treatment strategy for GBM [4]. However, due to the high metastatic rate, heterogeneity, and complexity of the GBM microenvironment, the vast majority of patients will be resistant to TMZ and relapse. Therefore, it is crucial and urgent to elucidate the mechanism of TMZ resistance and develop new chemotherapy drugs.

TTK Protein Kinase (TTK), also known as monopolar spindle 1 (Mps1) kinase, is a dual-specificity protein kinase that phosphorylates proteins on tyrosine, serine, and threonine [5, 6]. It has been widely believed that TTK participates in cell proliferation and division, and is essential for chromosome alignment at the centromere during mitosis and centrosome duplication [7]. In addition, accumulating evidence indicates that TTK is related to poor prognosis and malignant progression in gastric cancer [8], colon cancer [9], clear cell renal cell carcinoma [10], prostate cancer [11], breast cancer [12, 13], non-small-cell lung cancer [14], and medulloblastoma [15]. Recent research has shown that high TTK mRNA level correlates with earlier development of clinical symptoms, increased tumor aggressiveness, and poor outcome in patients with glioma [16]. The up-regulated TTK promotes proliferation and clonogenicity of glioma stem-like cells (GSCs) in vitro and in vivo [17].

TTK has also been implicated in mediating the radiosensitivity of glioma cells. Chen et al. demonstrate that hepatic leukemia factor (HLF)-mediated miR-132 directly suppresses TTK expression. TTK acts as an oncogene and contributes to the radioresistance of glioma cells [18]. Inhibition of TTK using NMS-P715 enhances radiosensitivity of human GBM cells through impairing DNA repair ability [19]. Additionally, TTK selective inhibitor MPS1-IN-3 has been proved to sensitize glioblastoma cells to antimitotic drugs [20]. However, the role of TTK in the TMZ resistance of GBM remains unclear.

In the current study, we demonstrated that knockdown of TTK sensitizes GBM cells to TMZ treatment, while overexpression of TTK promotes TMZ resistance of GBM. TTK specific inhibitors increased the sensitivity of GBM cells to TMZ. Moreover, TTK facilitated TMZ resistance through inducing autophagy in vitro and in vivo. Our study is beneficial to overcoming TMZ resistance and develops a novel therapeutic method for GBM.

## Methods

### Cell lines and cell culture

U87 MG cell lines were obtained from the American Type Culture Collection (ATCC, Manassas, VA, USA). U251 and HEK293T cell was purchased from the Cell Bank of the Chinese Academy of Sciences (Shanghai, China). U251, U87, and HEK293T were cultured in Dulbecco’s modified Eagle’s medium (DMEM) containing 10% fetal bovine serum (FBS, Gibco, Waltham, MA, USA) and 1% penicillin/streptomycin (B540732, Sangon Biotech, Shanghai, China) at 37 °C with 5% CO_2_.

### Antibodies and reagents

Antibodies for TTK (10381–1-AP), β-actin (20536–1-AP) were purchased from Proteintech (Wuhan, China); antibodies for poly [ADP-ribose] polymerase 1 (PARP1, 9532), cleaved caspase 3 (9664), and caspase 3 (9662) were obtained from Cell Signaling Technology (Danvers, MA, USA); antibodies for p62 (ab109012), Ki-67 (ab15580), and LC3B (ab192890) were obtained from Abcam (Cambridge, UK); BAY-1217389 (S8215), Chloroquine (CQ, S6999), Temozolomide (TMZ, S1237), and Thiazolyl Blue (MTT, S6821) were acquired from Selleck Chemicals (Houston, TX, USA). CFI-402257 (A12037) was acquired from Adooq Bioscience (Irvine, CA, USA).

### Western blot

U251 and U87 cells were lysed using the RIPA Lysis Buffer (AR0105–100, Boster, Pleasanton, CA, USA) containing 1 mM phenylmethylsulfonyl fluoride (PMSF). After being centrifuged, the protein concentration was measured with a bicinchoninic acid (BCA) protein assay kit (C503021, Sangon Biotech). The proteins were electrophoresed on 12–15% SDS–PAGE gels and then transferred onto polyvinylidene fluoride (PVDF) membranes. After that, the membranes were incubated with 5% skimmed milk for 1 h at room temperature (RT), indicated primary antibodies overnight at 4 °C, and then with the appropriate secondary antibodies for 1 h at RT in turn. The blots were imaged with an enhanced chemiluminescence detection system and GE Amersham Imager AI680 (GE, Chicago, IL, USA). β-actin was used as a loading control. Data were analyzed by ImageJ software (US National Institutes of Health).

### Colony formation assay

Cells (800 cells per well) were seeded in the 6-well plate and incubated for the indicated time. Then, the colonies were stained with 0.1% crystal violet which dissolved in methanol. Colonies with > 50 cells per colony were counted with ImageJ software.

### MTT assay

Cells were seeded into a 96-well plate at a density of 3000 cells per well. After treatment, the culture medium was replaced by MTT solution and incubated for 4 h at 37 °C. After that, the medium was gently removed, and 200 μL dimethyl sulfoxide (DMSO) was added into each well. Next, the plate was put on a shaker and gently vortexed for 10 min at RT in the dark to fully dissolve the crystals. The absorbance values were measured at 490 nm using a microplate reader (ThermoFisher Scientific, Waltham, MA, USA).

### Plasmid construction

The PLKO.1 TTK shRNA1 (TRCN0000006358) and PLKO.1 TTK shRNA2 (TRCN0000011012) were purchased from Sigma-Aldrich (St. Louis, MO, USA). PCMV TTK plasmids were generated by Genechem (Shanghai, China). PLKO.1 and PCMV empty plasmids were used as the negative controls. All of the vectors were verified by DNA sequencing. Lentivirus particles were produced in HEK293T cells packaged by psPAX2 and pMD2.G using Lipofectamine 2000 reagent (11,668,019, ThermoFisher Scientific) according to the manufacturer’s protocol. To acquire cells with stable TTK knockdown or overexpression, cells were incubated with lentivirus for 24 h and then selected for 3–7 days in the corresponding medium containing 2 μg/ml puromycin (P8833, Sigma-Aldrich).

### Immunohistochemistry staining (IHC)

Tumor nodules were fixed using formalin, embedded in paraffin, and sectioned at a 5 μm thickness slide. The tissue sections were deparaffinized with xylene, rehydrated using graded ethanol, and washed with water. IHC staining was performed using the One-Step IHC Assay Kit (KGOS300, KeyGEN, Nanjing, China) according to the manufacturer’s protocol. In brief, after antigen retrieval using ethylenediaminetetraacetic acid (EDTA) buffer, the slides were incubated with 3% H_2_O_2_ to eliminate endogenous peroxidase activity. Then, the slides were blocked, and incubated with primary antibodies overnight at 4 °C, biotinylated secondary antibody for 20 min at 37 °C, and horseradish peroxidase (HRP) labeled tertiary antibody for 20 min at 37 °C in turn. After that, the slides were stained with 3,3′-diaminobenzidine (DAB) and counterstained with hematoxylin. At last, the slides were dehydrated, incubated in xylol, and mounted. The positive ratio of cells was calculated by two trained pathologists according to the intensity of staining. At least five randomly selected, nonadjacent, nonoverlapping areas at 200× were evaluated and the mean value was adopted.

### Immunofluorescence staining

Cells transfected with TTK or not were fixed with 4% paraformaldehyde for 10 min at RT. Then, the cells were permeabilized using Triton X-100 for 2 min and blocked with goat serum for 30 min at RT. Afterward, the cells were incubated with LC3B primary antibody (1:100) for 18 h at 4 °C and FITC secondary antibody (1:200) for 1 h at 37 °C. The nuclei were labeled with DAPI. The Zeiss LSM 800 (Carl Zeiss, Jena, Germany) was used for fluorescence detection.

### Tumor formation assay in nude mice

All of the animal research was carried out under the approval of the Animal Research Committee of The First Affiliated Hospital of the University of Science and Technology of China. Male nude mice (4–6 weeks old, 18–20 g) were purchased from the Viewsolid Biotechnology (Beijing, China) and housed in a pathogen-free environment. U251 cells (5 × 10^6^) with TTK overexpression or not were subcutaneously injected into the nude mouse. The size of the tumor was measured by vernier calipers and calculated as the length × width^2^ × 0.5. When the tumor volumes reached approximately 100 mm^3^, the animals were randomly divided into 3 groups with 6 animals per group and the treatments were initiated by intraperitoneal injection of temozolomide and/or CQ. Two weeks post injection, the mice were sacrificed to determine the tumor volumes and photographed.

### Statistical analysis

All experiments were repeated at least three times. The results were expressed as mean ± standard error of the mean (SEM) and were analyzed using SPSS 26.0 software (IBM, Chicago, IL, USA). The comparison between the two groups was conducted with Student’s t-test. For comparisons among multiple groups, one-way analysis of variance (ANOVA) with Fisher’s least significant difference (LSD) test was used. Images were processed using GraphPad Prism 9.00 (GraphPad Software, La Jolla, CA, USA) and Adobe Photoshop CC 22.0 (Adobe, San Jose, CA, USA). *p* < 0.05 was considered as statistically significant.

## Results

### Knockdown of TTK sensitizes GBM cells to TMZ

**Fig. 1:**
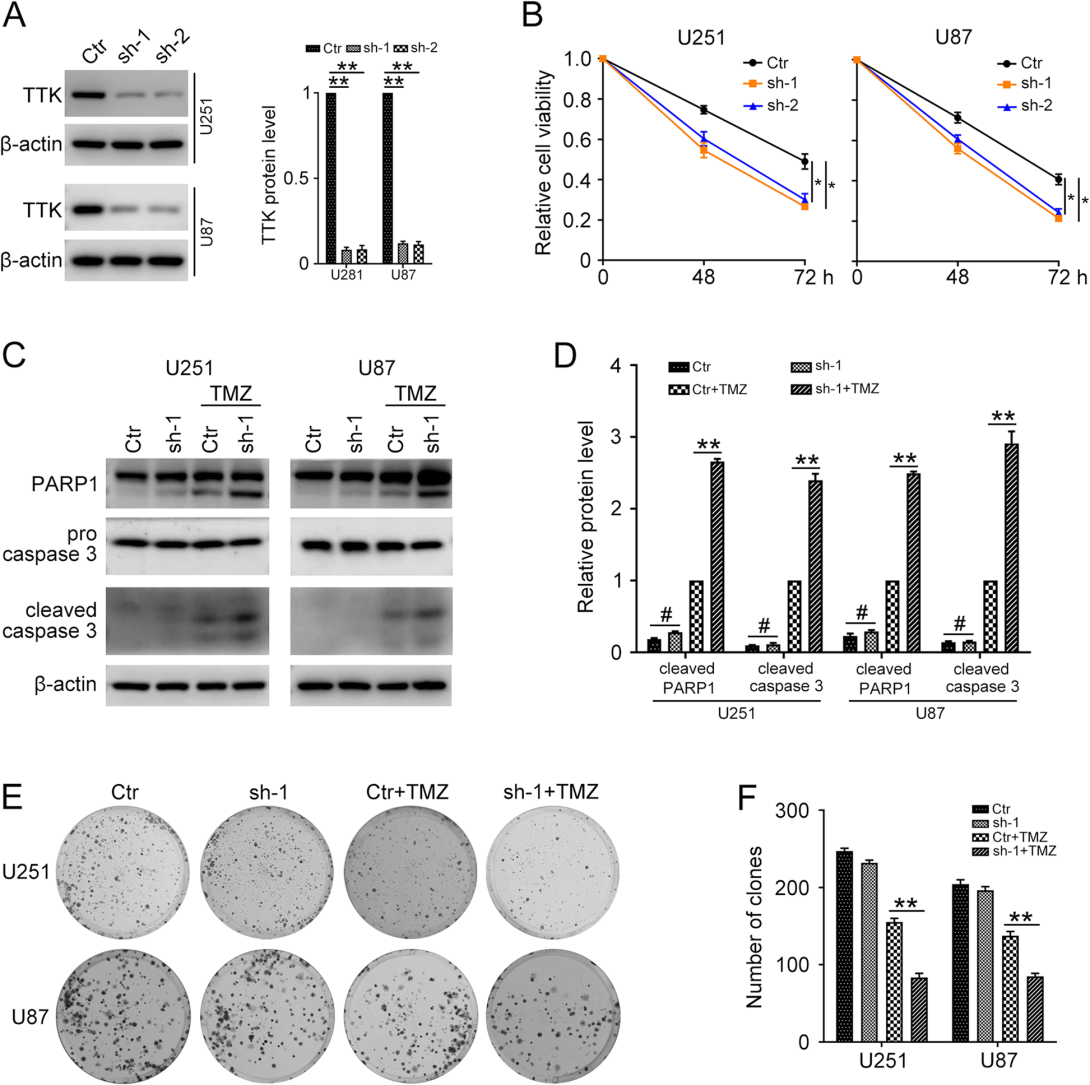
Knockdown of TTK increased temozolomide sensitivity of GBM cells. U251 and U87 cells were stably transfected with PLKO.1 (Ctr), TTK shRNA1 (sh-1), or TTK shRNA2 (sh-2), then treated with 100 μM temozolomide (TMZ) for 48 and 72 h. **A** Western blot was used to determine the protein levels of TTK and β-actin. **B** MTT assay was conducted to analyze the cell viability. **C** U251 and U87 cells transfected with PLKO.1 (Ctr) or TTK shRNA1 (sh-1) were treated with 100 μM TMZ for 72 h, western blot was performed to determine the protein levels of cleaved PARP1, cleaved caspase 3, and β-actin. **D** Quantification of relative protein levels in (C). **E** Clonogenic assay was performed to assess the colony formation efficiency of U251 and U87 cells stably transfected with PLKO.1 (Ctr) or TTK shRNA1 (sh-1) with or without 20 μM TMZ treatment. **F** Quantification of the number of clones in (E). The uncropped blots are displayed in Additional file 1. (Data are mean ± SEM, #*p* > 0.05, *p < 0.05, **p < 0.01, *n* = 3)

To determine the role of TTK in the TMZ sensitivity of GBM cells, two independent TTK shRNAs (shRNA1 and shRNA2) were stably transfected into U251 and U87 cells. The knockdown efficiency of TTK was confirmed using western blot (Fig. 1A). Then, U251 and U87 cells with TTK knockdown were incubated with 100 μM temozolomide (TMZ) for 48 or 72 h. The MTT assay showed that knockdown of TTK significantly decreased the cell viability of U251 and U87 cells treated with TMZ compared with control groups (Fig. 1B). Next, western blot was performed to investigate the apoptosis in U251 and U87 cells with TTK knockdown in response to TMZ. The results demonstrated that cells with TTK knockdown showed an increased level of apoptotic protein cleaved caspase 3 and cleaved PARP1 (Fig. 1C and D). Moreover, the clonogenic assay also showed that knockdown of TTK impaired the colony forming ability of GBM cells upon TMZ treatment (Fig. 1E and F). Therefore, these results suggested that knockdown of TTK increases the TMZ sensitivity of GBM cells.

### Overexpression of TTK promotes TMZ resistance in glioma cells

**Fig. 2:**
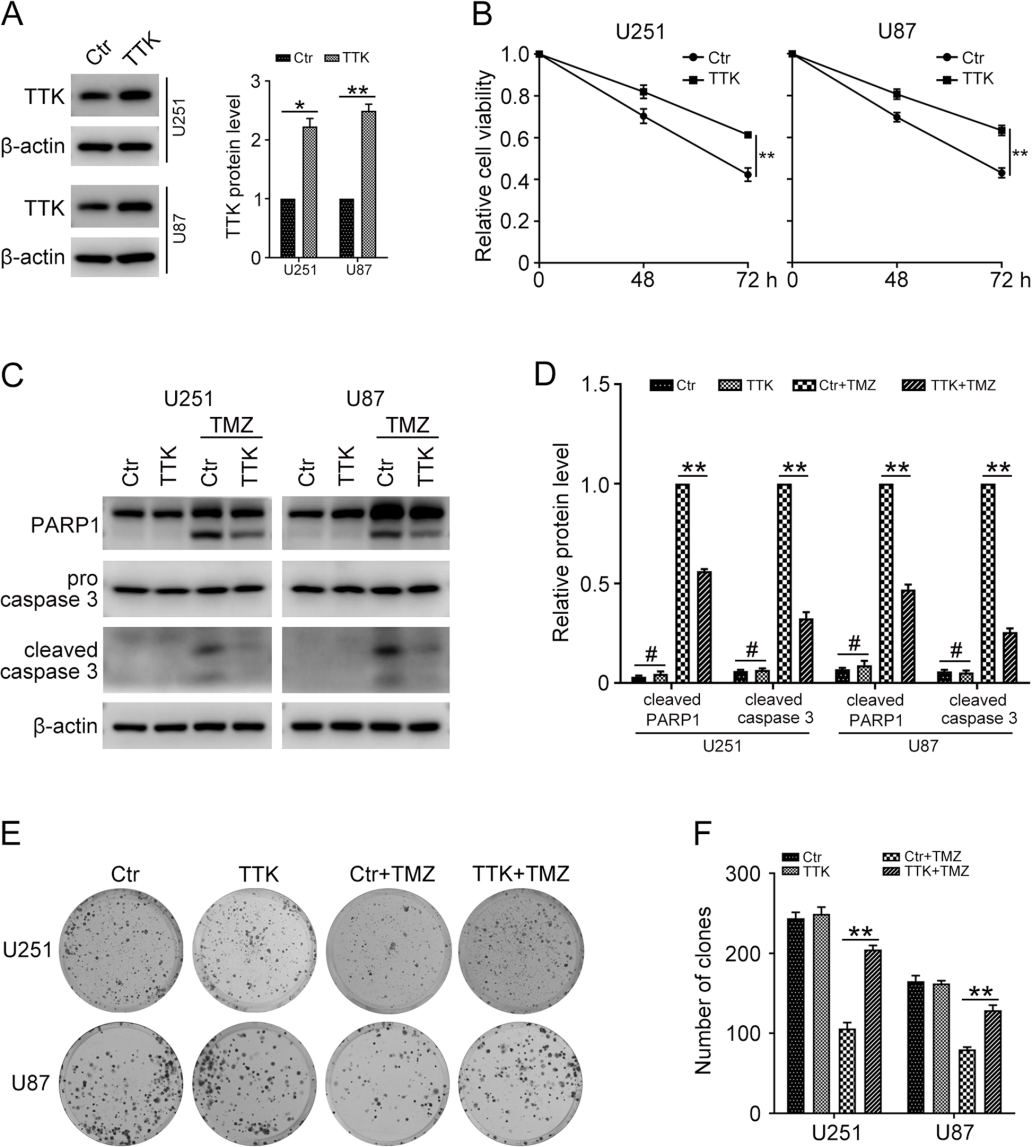
Overexpression of TTK promoted TMZ resistance in GBM cells. U251 and U87 cells were stably transfected with PCMV (Ctr) or PCMV TTK (TTK), then treated with 100 μM temozolomide (TMZ) for 48 and 72 h. **A** Western blot was used to determine the protein levels of TTK and β-actin. **B** MTT assay was conducted to analyze the cell viability. **C** U251 and U87 cells transfected with PCMV (Ctr) or PCMV TTK (TTK) were treated with 100 μM TMZ for 72 h, western blot was performed to determine the protein levels of cleaved PARP1, cleaved caspase 3, and β-actin. **D** Quantification of relative protein levels in (C). **E** Clonogenic assay was performed to assess the colony formation efficiency of U251 and U87 cells stably transfected with PCMV (Ctr) or PCMV TTK (TTK) with or without 20 μM TMZ treatment. **F** Quantification of the number of clones in (E). The uncropped blots are displayed in Additional file 1. (Data are mean ± SEM, #*p* > 0.05, *p < 0.05, **p < 0.01, *n* = 3)

To better clarify the function of TTK in the TMZ resistance of GBM cells, we overexpressed TTK by transfecting the PCMV TTK plasmid into U251 and U87 cells. Western blot showed that the protein level of TTK was dramatically increased in cells transfected with PCMV TTK (Fig. 2A). Correspondingly, overexpression of TTK increased the cell viability in U251 and U87 cells with TMZ treatment (Fig. 2B). Additionally, overexpression of TTK reduced the protein level of cleaved caspase 3 and cleaved PARP1 induced by TMZ treatment (Fig. 2C and D). The clonogenic assay also confirmed that U251 and U87 cells with TTK overexpression possessed stronger colony forming ability (Fig. 2E and F). Taken together, our results indicated that overexpression of TTK conferred TMZ resistance in GBM cells.

### TTK inhibitors significantly decrease glioma cell viability

**Fig. 3:**
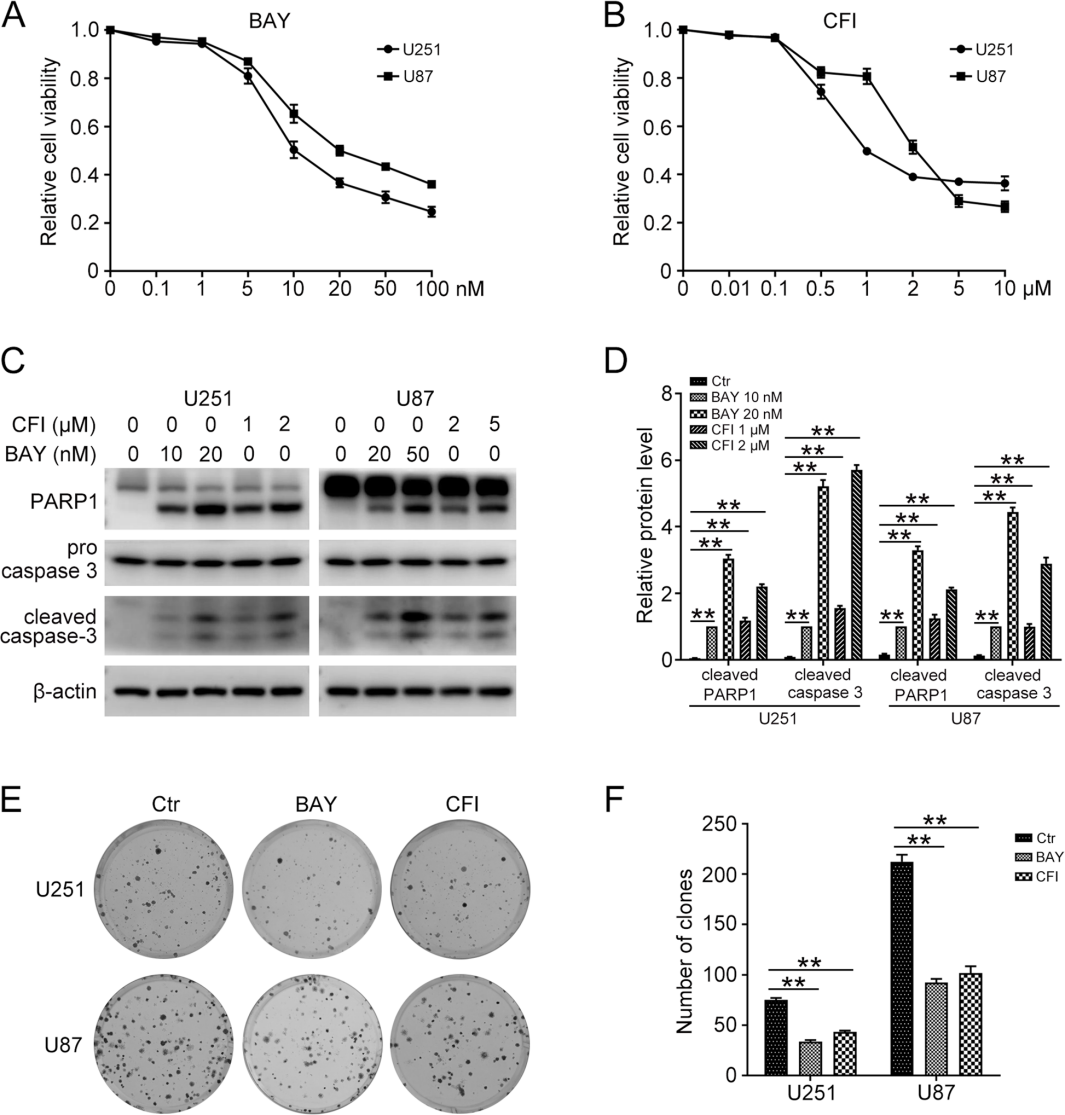
TTK inhibitors inhibited cell proliferation of GBM cells. **A** U251 and U87 cells were treated with different concentrations (0, 0.1, 1, 5, 10, 20, 50, 100 nM) of BAY-1217389 (BAY) for 48 h. MTT assay was used to detect the cell viability. **B** U251 and U87 cells were treated with different concentrations (0, 0.01, 0.1, 0.5, 1, 2, 5, 10 μM) of CFI-402257 (CFI) for 48 h. MTT assay was used to detect the cell viability. **C** U251 and U87 cells were treated with BAY (U251, 10 and 20 nM; U87, 20 and 50 nM) or CFI (U251, 1 and 2 μM; U87, 2 and 5 μM) for 48 h, western blot was performed to determine the protein levels of cleaved PARP1, cleaved caspase 3, and β-actin. **D** Quantification of relative protein levels in (C). **E** Clonogenic assay was performed to assess the colony formation efficiency of U251 and U87 cells with BAY (U251, 5 nM; U87, 10 nM) or CFI (U251, 0.5 μM; U87, 1 μM) treatment. **F** Quantification of the number of clones in (E). The uncropped blots are displayed in Additional file 1. (Data are mean ± SEM, *p < 0.05, **p < 0.01, *n* = 3)

To examine the effect of TTK inhibitors on the proliferation of GBM cells, U251 and U87 cells were treated with a series of concentrations of BAY-1217389 (BAY) or CFI-402257 (CFI) for 48 h. Results from the MTT assay demonstrated that different concentrations of BAY and CFI exhibites a strong growth suppressive effect on U251 and U87 cells in a dose dependent manner (Fig. 3A and B). BAY and CFI have been reported to induce cell apoptosis in a variety of cancer cells. Next, we turned to explore whether BAY and CFI could increase the apoptosis in GBM cells. Western blot results showed that the protein levels of cleaved caspase 3 and cleaved PARP1 were dramatically increased under BAY and CFI treatment in U251 and U87 cells (Fig. 3C and D). Moreover, compared with the control group, BAY and CFI stimulation significantly inhibited the colony forming abilities of GBM cells (Fig. 3E and F). Thus, TTK specific inhibitors significantly inhibited GBM cell proliferation.

### TTK inhibitors increase TMZ sensitivity in GBM cells

**Fig. 4:**
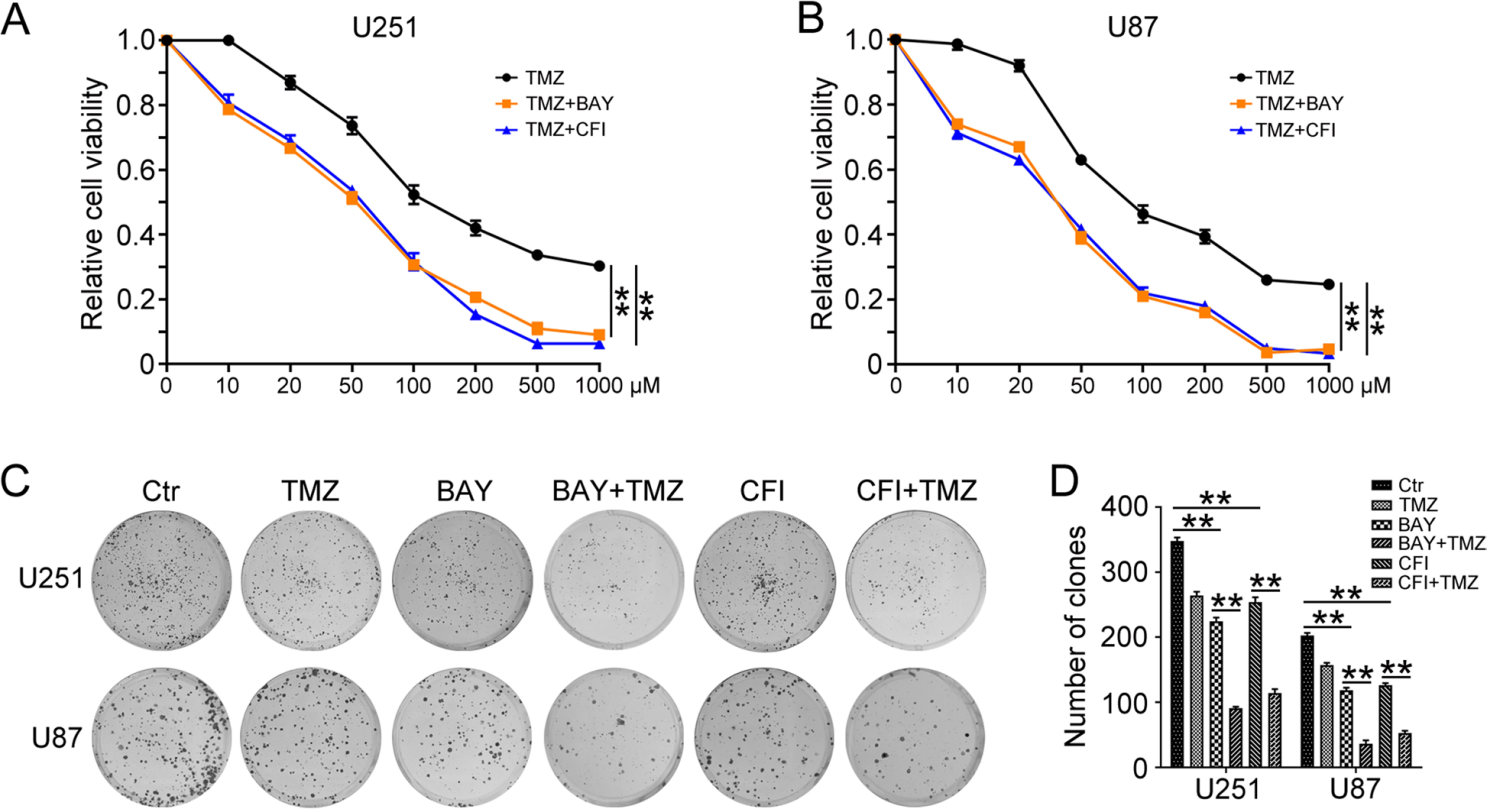
TTK inhibitors increased the efficiency of TMZ in GBM cells. **A** U251 and (**B**) U87 cells were treated with or without TMZ (10, 20, 50, 100, 200, 500,1000 μM), BAY (U251, 10 nM; U87, 20 nM), and CFI (U251, 1 μM; U87, 2 μM) for 48 h. The MTT assay was used to detect cell viability. **C** U251 and U87 cells were treated with or without TMZ (20 μM), BAY (U251, 5 nM; U87, 10 nM), and CFI (U251, 0.5 μM; U87, 1 μM). Clonogenic assay was performed to assess the colony formation efficiency. (D) Quantification of the number of clones in (C). (Data are mean ± SEM, **p < 0.01, *n* = 3)

Next, we tried to investigate whether TTK inhibitors could enhance TMZ sensitivity in glioma cells. U251 and U87 cells treated with different concentrations of TMZ were incubated with BAY and CFI for 48 h, and MTT assay was performed to detect cell viability. As shown in Fig. 4A and B, compared with TMZ alone treated group, BAY and CFI in combination with TMZ treatment showed a dramatic decrease of cell viability in U251 and U87 cells. Moreover, the clonogenic assay showed that the colony forming suppressive effect of TMZ and BAY/CFI on GBM cancer cells was significantly better than that of TMZ alone (Fig. 4C-D). Collectively, these findings indicated that TTK inhibitors enhances the lethal effect of TMZ in GBM cells. Combined TTK inhibitors with TMZ might possess a bright future in the clinical treatment of GBM.

### TTK promotes TMZ resistance through inducing autophagy

**Fig. 5:**
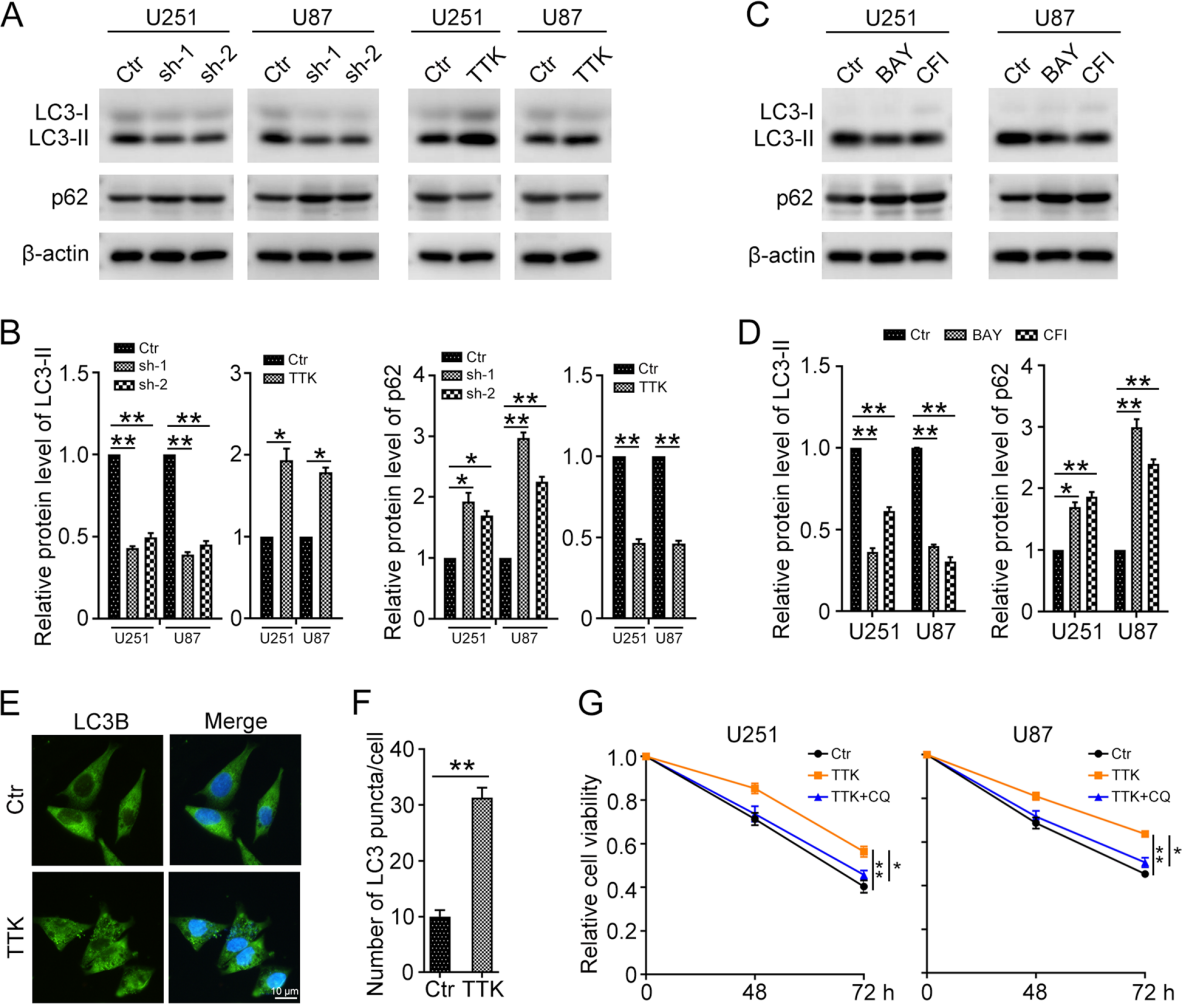
TTK prompted TMZ resistance through inducing autophagy in GBM cells. **A** Western blot was used to determine the protein levels of LC3-I, LC3-II, p62, and β-actin in U251 and U87 cells transfected with PLKO.1 (Ctr), TTK shRNA1 (sh-1), TTK shRNA2 (sh-2), PCMV (Ctr), or PCMV TTK (TTK). **B** Quantification of relative protein levels in (A). **C** Western blot was used to determine the protein levels of LC3-I, LC3-II, p62, and β-actin in U251 and U87 treated with BAY (U251, 10 nM; U87, 20 nM), and CFI (U251, 1 μM; U87, 2 μM) for 12 h. **D** Quantification of relative protein levels in (C). **E** Immunofluorescence staining was used to detect LC3B puncta in U251 cells transfected with PCMV (Ctr) or PCMV TTK (TTK). Scale bar: 10 μm. **F** Quantification of the number of LC3 puncta/cell. **G** U251 and U87 cells transfected with PCMV or PCMV TTK were treated with TMZ (100 μM) or/and CQ (50 μM) for 48 h or 72 h. The MTT assay was used to detect cell viability. The uncropped blots are displayed in Additional file 1. (Data are mean ± SEM, *p < 0.05, **p < 0.01, *n* = 3)

The cellular autophagy process has been shown to play a vital role in the chemoresistance of several cancers [21, 22]. Next, western blot was performed to explore the autophagy levels in U251 and U87 cells with TTK knockdown and overexpression. The results showed that knockdown of TTK significantly decreases the protein level of autophagy marker LC3-II, and increased the expression of autophagy substrate p62 compared with those in the control group. Correspondingly, overexpression of TTK increased the LC3-II level and decreased p62 level in U251 and U87 cells (Fig. 5A and B). TTK inhibitors could also significantly inhibit autophagy level, as indicated by the downregulated LC3-II level and upregulated p62 level in GBM cells (Fig. 5C and D). Meanwhile, immunofluorescence staining of LC3B showed an increased number of LC3B puncta in U251 cells transfected with PCMV TTK (Fig. 5E and F). To clarify the effect of autophagy on TTK-induced drug resistance, CQ was added to inhibit the autophagy process. The MTT assay showed that the TMZ resistant effect of TTK was reversed in U251 and U87 cells treated with CQ (Fig. 5G). Therefore, these results suggested that overexpression of TTK enhances TMZ resistance via inducing autophagy in GBM cells.

### TTK promotes TMZ resistance through autophagy in vivo

**Fig. 6:**
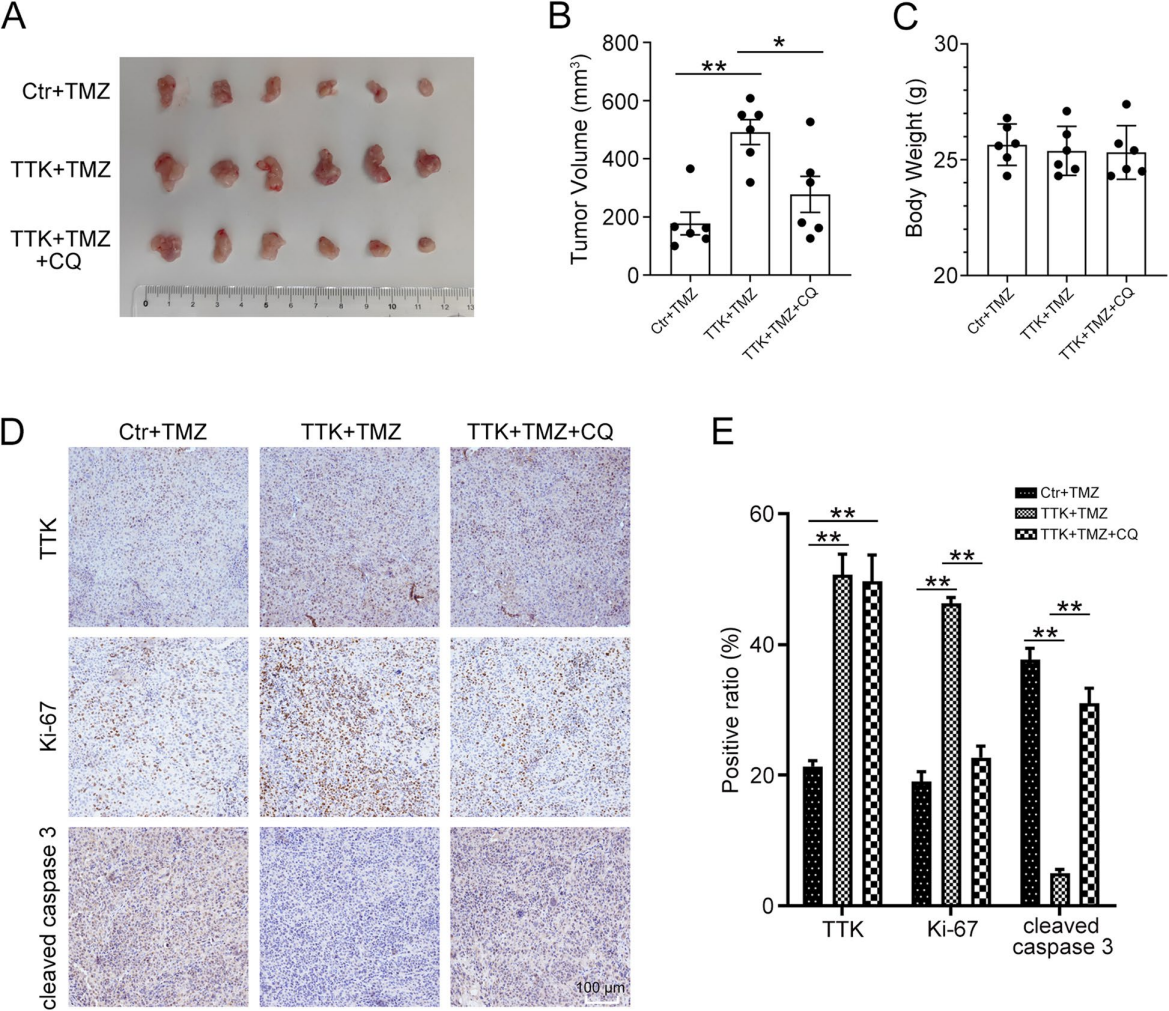
Overexpression of TTK induced TMZ resistance through autophagy in vivo. About 5 × 10^6^ U251 cells stably transfected with PCMV (Ctr) or PCMV TTK (TTK) were subcutaneously injected into the nude mice. When the tumor volume reached about 100 mm^3^, the mice then received intraperitoneal injection of TMZ (50 mg/kg, every day) or/and CQ (30 mg/kg, every day) for 2 weeks. **A** Representative image of gross tumors from the mice. **B** Tumor volumes of each group were measured. **C** The mouse body weight of each group. **D** Representative images of IHC staining of TTK, Ki-67, and cleaved caspase 3 in tumor tissues. Scale bar: 100 μm. **E** Quantification of the positive ratio of TTK, Ki-67, and cleaved caspase 3 in (D). (Data are mean ± SEM, *p < 0.05, **p < 0.01, *n* = 6)

Given the in vitro findings, we attempted to investigate the effect of TTK-induced autophagy on TMZ sensitivity in the mouse xenograft model. U251 cells with TTK overexpression or not were subcutaneously injected into the nude mice. Two weeks later, the tumor-bearing mice received intraperitoneal injection of TMZ or/and CQ for 15 days. The tumor volumes in cells transfected with PCMV TTK were significantly larger than those in cells transfected with PCMV control, indicating the TMZ resistant function of TTK in vivo. However, the administration of CQ reversed the effect of TTK overexpression (Fig. 6A-C). IHC staining showed that overexpression of TTK increases the protein level of Ki-67 and decreased the level of cleaved caspase 3, while inhibition of autophagy using CQ exhibited the opposite effect (Fig. 6D and E). Thus, our results indicated that TTK confers TMZ resistance through autophagy in the mouse xenograft model.

## Discussion

As a dual-specificity protein kinase, TTK has been first proven to play a key role in spindle assembly checkpoint (SAC), and subsequent studies find that TTK is also involved in other processes, such as centrosome duplication, DNA damage response, organ development, tumor progression, chemoresistance, and radioresistance [23]. The expression of TTK is almost undetectable in normal tissues except the testis and placenta. However, TTK is commonly expressed in a variety of tumor tissues, including glioma, making it a potential target for cancer therapies [24, 25]. Inhibition of TTK activity sensitizes basal-like breast cancer to radiation therapy by destroying DNA repair efficiency [26]. TTK is overexpressed in cisplatin-resistant ovarian cancer cells and ovarian cancer patients with acquired resistance to cisplatin. TTK knockdown increases the sensitivity of cisplatin-resistant ovarian cancer cells to cisplatin via the PI3K/AKT signaling pathway [27]. Inhibition of TTK alters cell-cycle progression and exacerbates centrosome abnormalities, thus promoting the radiosensitivity of liver cancer cells in a p21-mediated manner [28]. In this study, we demonstrated that knockdown of TTK decreases the cell viability and increases the apoptosis in GBM cells with TMZ treatment. However, overexpression of TTK reached the opposite conclusions. Overexpression of TTK also decreased the sensitivity of GBM cells to TMZ in the mouse model. Therefore, TTK is a promising therapeutic target and molecular biomarker for effective GBM treatment. Targeted inhibition of TTK may have a bright clinical prospect in the near future.

A variety of TTK inhibitors have been developed to improve the effectiveness of tumor therapy or overcome drug resistance. Up to now, several studies have yielded encouraging results about the anti-proliferative activity of TTK inhibitors in combination with chemotherapy or radiotherapy [29]. CFI-402257, a selective orally bioavailable inhibitor of TTK, displays strong anti-tumor efficiency in combination with cisplatin/pemetrexed in malignant mesothelioma (MM) [30]. Another TTK inhibitor, NTRC0066–0, inhibits the proliferation via inducing chromosome missegregation in cell lines and in mice. NTRC0066–0 combined with a therapeutic dose of docetaxel results in extended tumor remission and doubled survival time in triple negative breast cancer (TNBC) [31]. TTK inhibitors BAY 1161909 and BAY 1217389 in combination with antimitotic cancer drugs achieve obvious enhancement effects over paclitaxel or TTK inhibitor monotreatment through abrogating SAC in a variety of xenograft models [32]. NMS-P715 has been shown to sensitize GBM cells to radiation therapy through impairing DNA double-strand breaks (DSB) and induction of postradiation mitotic catastrophe [19]. Here, we reported that TTK inhibitors BAY 1217389 and CFI-402257 induces cell apoptosis and significantly decreases the proliferation of GBM cells dose-dependently. Additionally, these two inhibitors effectively increased the TMZ sensitivity in GBM. Combined TTK inhibitors and TMZ induced greater cell apoptosis and showed a stronger anti-proliferative effect than the control group. Thus, these results highlight the potential value of TTK inhibitors as therapeutic options to overcome TMZ resistance in GBM. The combination of TMZ with TTK inhibitors may have an attractive therapeutic effect in clinical treatment.

Autophagy is a conserved molecular pathway that eliminates damaged and defective cellular materials, including nucleic acids, proteins, and organelles, via lysosome-mediated degradation [33]. Autophagy plays vital roles in several cellular functions, including tumorigenesis, tumor-stroma interactions, and tumor microenvironment [34]. It is believed that autophagy protects the cancer cells from various chemotherapeutics by providing recycled nutrients and energy to cells [35]. The previous study has proved that TMZ induces autophagy in malignant glioma cells and suppression of autophagy using CQ or Bafilomycin A1 (Baf A1) can increase the therapeutic efficacy of TMZ [36]. The relationship between TTK and autophagy in GBM cells remains obscure. In the current study, we demonstrated that TTK knockdown impaires the autophagy and TTK overexpression promoted autophagy in GBM cells. Inhibition of TTK using specific inhibitors also markedly decreased cellular autophagy level. Suppression of autophagy using CQ could counteract the TMZ resistance induced by TTK overexpression in vitro and in a xenograft model. Our results suggest that blocking autophagy could be a promising approach to overcome TMZ resistance in GBM cells with high TTK expression.

## Conclusions

In this study, we demonstrated that knockdown of TTK sensitizes GBM cells to TMZ, while overexpression of TTK promotes TMZ resistance in GBM. Two specific TTK inhibitors dramatically decreased GBM cell viability and enhanced the growth suppressive effect of TMZ. Mechanistically, TTK contributed to TMZ resistance through inducing autophagy in vitro and in vivo. Our findings help to reveal the mechanism of TMZ resistance and provide potential solutions for overcoming TMZ resistance in clinical practice.

## Supplementary Information

**Additional file 1.**

## Data Availability

All data generated or analyzed during this study are included in this published article and its supplementary information files.

## Abbreviations

ATCC: American Type Culture Collection
BCA: Bicinchoninic acid
CQ: Chloroquine
DAB: 3,3′-diaminobenzidine
DMSO: Dimethyl sulfoxide
EDTA: Ethylenediaminetetraacetic acid
FBS: Fetal bovine serum
GBM: Glioblastoma multiforme
HLF: Hepatic leukemia factor
IHC: Immunohistochemistry
Mps1: Monopolar spindle 1
PARP1: Poly [ADP-ribose] polymerase 1
PMSF: Phenylmethylsulfonyl fluoride
PVDF: Polyvinylidene fluoride
RT: Room temperature
SAC: Spindle assembly checkpoint
SEM: Standard error of the mean
TMZ: Temozolomide
TTK: TTK Protein Kinase
