# Supplementary material for: TTK Protein Kinase promotes temozolomide resistance through inducing autophagy in glioblastoma
Source: BMC Cancer. 2022 Jul 18;22:786. doi: 10.1186/s12885-022-09899-1 (PMC9290216; doi:10.1186/s12885-022-09899-1)

The uncropped gels/blots of Figure 1.


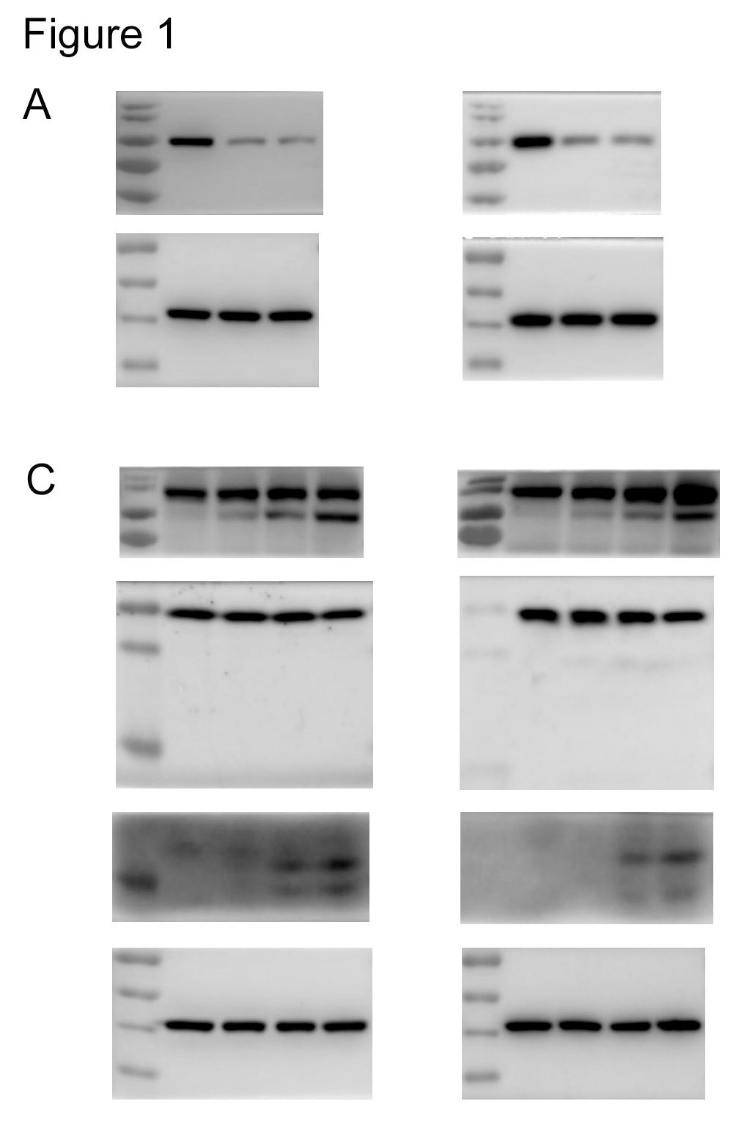


The uncropped gels/blots of Figure 2.


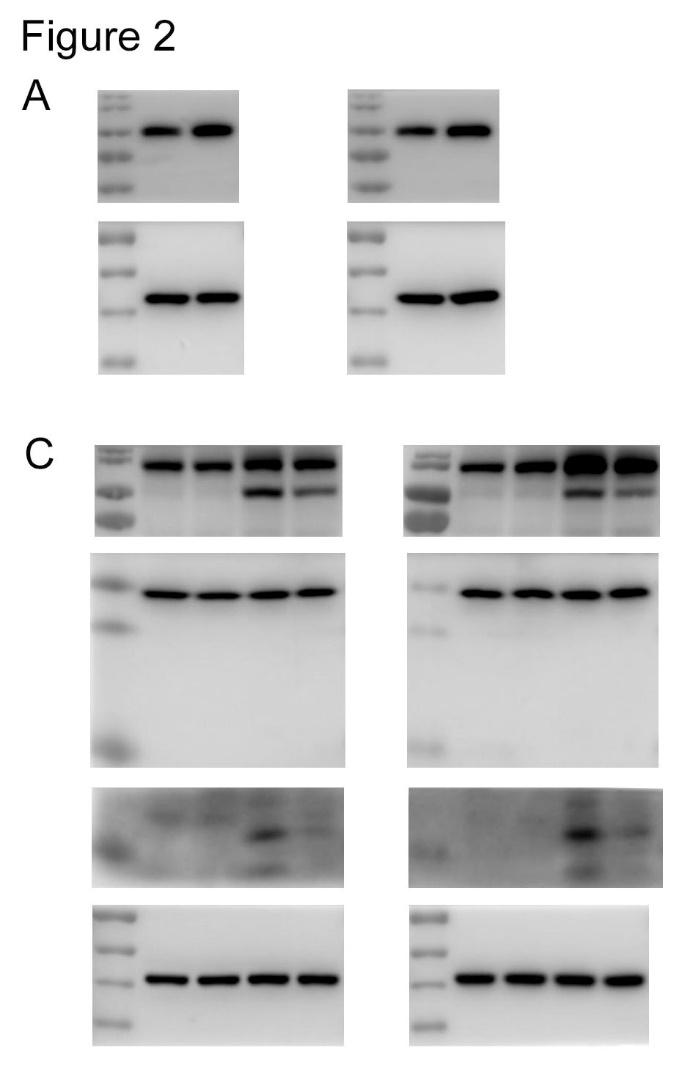


The uncropped gels/blots of Figure 3.


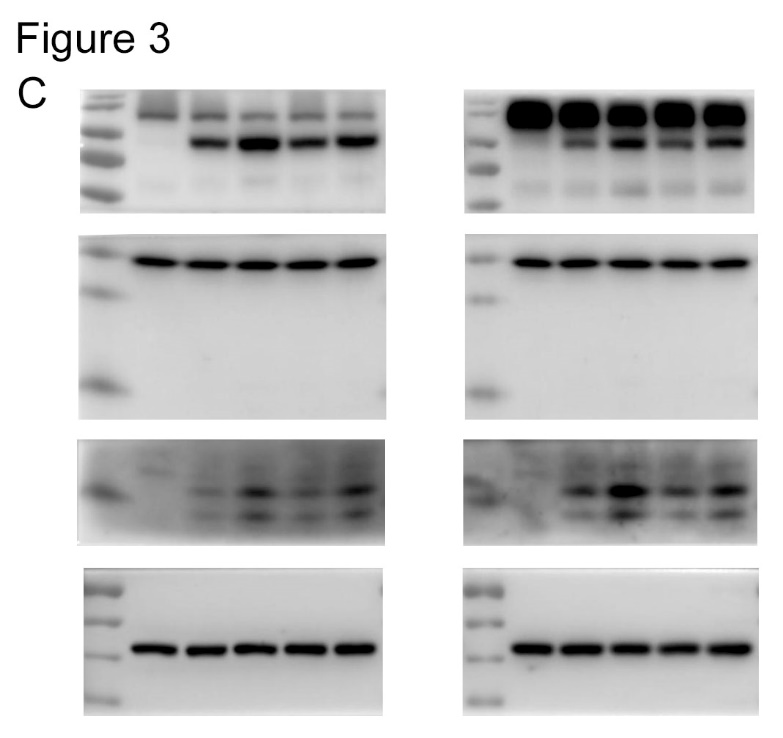


The uncropped gels/blots of Figure 5.


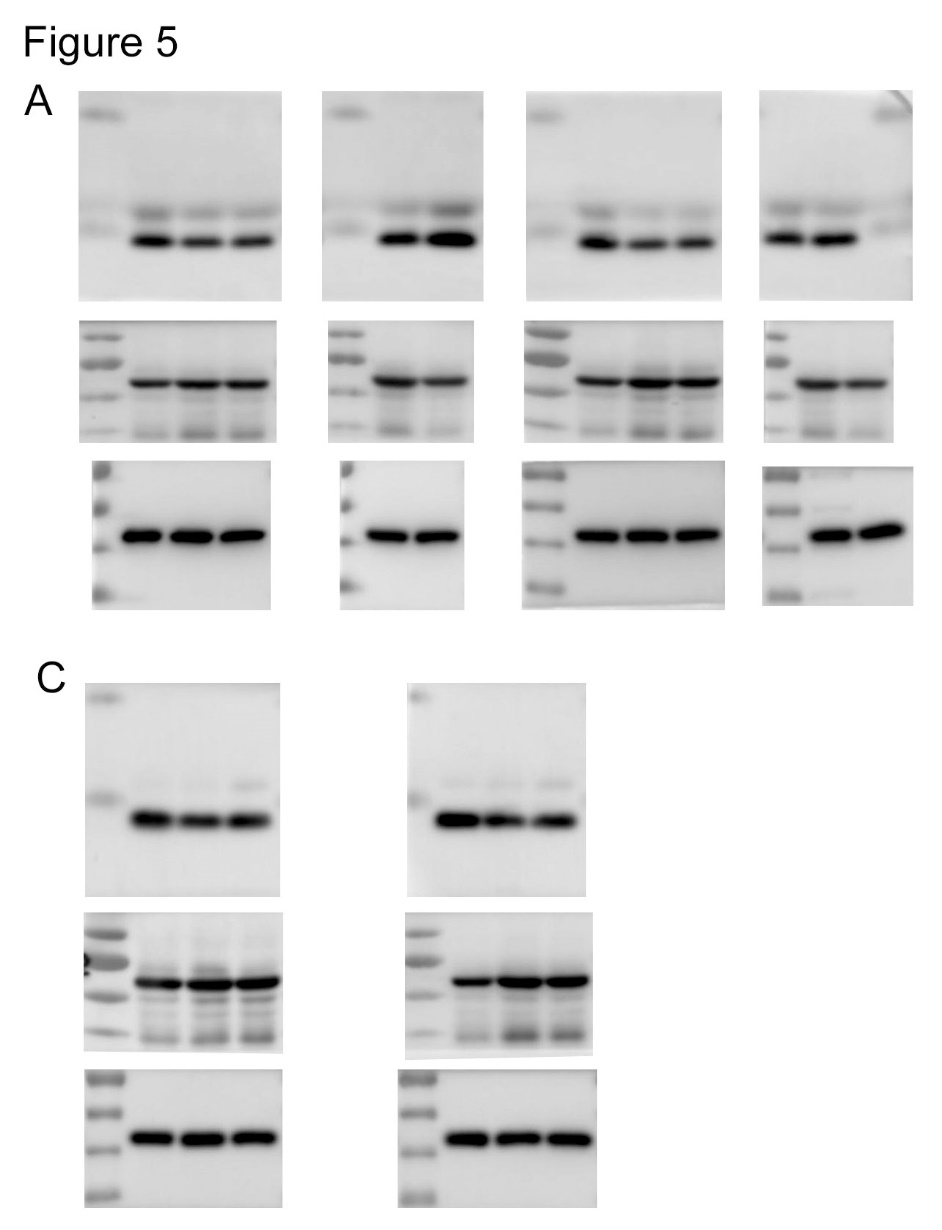

Supplement: Supplementary file 1 — Additional file 1. [file 12885_2022_9899_MOESM1_ESM.docx]
